# Scaled testing for COVID-19 needs community involvement

**DOI:** 10.7189/jogh.11.03033

**Published:** 2021-03-01

**Authors:** Emily R Adams, Dawit Wolday, Claudia M Denkinger, Sanjeev Krishna, Tobias F Rinke de Wit

**Affiliations:** 1 Centre for Drugs and Diagnostics, Liverpool School of Tropical Medicine, Liverpool, UK; 2 Mekelle University College of Health Sciences, Mekelle, Ethiopia; 3 Division of Tropical Medicine, Center of Infectious Diseases, Heidelberg University Hospital, Heidelberg, Germany; 4 St George’s University of London, London, UK; 5 Joep Lange Institute, Amsterdam, the Netherlands; 6 PharmAccess Foundation, Amsterdam, the Netherlands

With limited vaccine coverage and in the absence of specific therapeutics, the options to curb the COVID-19 pandemic today are mostly infection prevention measures and diagnostic tests to identify infected individuals rapidly, followed by isolation and contact tracing. The past months have seen significant worldwide scale-up of testing through quantitative PCR (qPCR) detecting nucleic acid of SARS-CoV-2. However, with the current resurgence of cases in Europe and elsewhere, qPCR demand far exceeds capacity. Moreover, qPCR is expensive, requires sophisticated laboratories, well-trained staff, and extensive logistics. Capacity deficits are aggravated in LMICs (Lower and Middle-Income Countries) with rampant shortages of tests kits, qualified staff, and laboratories, compromising clinical utility. Africa CDC reports 23 countries with proxy PCR coverage of <5000 tests/million population [1]. Alternative novel molecular diagnostics such as LAMP, DNAnudge and LAMPore remain widely unscalable. Moreover, even qPCR has its limitations in detecting non-infectious traces of viral RNA [2] and false negativity issues [3]. We conclude that molecular testing for COVID-19 is unfit for reaching scale at the community level.

Rapid diagnostic tests detecting viral antigen (AgRDT), which can be delivered at the point of care (POC) provide a relevant alternative approach for direct measurement of the virus. AgRDTs can be performed manually, in 15-20 minutes, with the option to digitize return of test results through simple mobile applications. AgRDTs are reportedly less sensitive than the ‘gold standard’ qPCR [4], but they can be useful to alleviate the pressure on molecular testing. Many countries are currently including AgRDTs into their testing algorithms. However, these algorithms are still reliant on the capacity of (public) health professionals, who are increasingly scarce, overworked and often in period of self-isolation themselves. We reason for COVID-19 strategies that can directly reach out to communities to provide options of self-testing and thus a more prominent role in scaled testing, alleviating the overburdened health sector.

**Figure:**
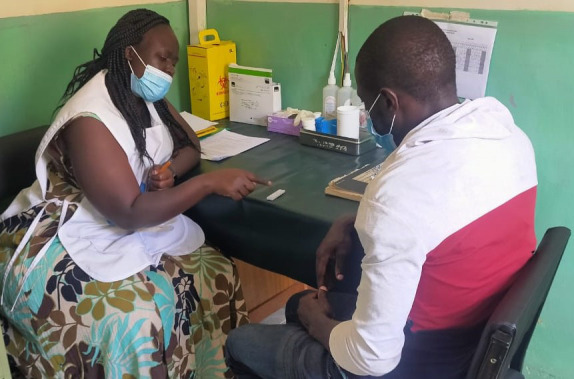
Photo: Kisumu community counsellor discussing COVID RDT result with client (from Emmanuel Milimo’s own collection, used with permission).

## CHALLENGES IN EUROPE

Over the first half of 2020, Europe implemented wide scale responses to COVID-19 including national lockdowns, social distancing and hygiene measures, travel restrictions, contact tracing and quarantine. Such interventions have increasingly met with resistance in societies, particularly due to their associated large social and economic harms. Europe is currently experiencing a ‘second surge’ of COVID-19. Hospitals are again experiencing serious capacity challenges. In many settings the COVID-19 diagnostic testing system is overloaded and priority groups (health care staff, schoolteachers, police) who require frequent testing, are consistently underserved. AgRDTs are increasingly applied as supplementary means, but still mostly in the context of professional health care delivery, and in the UK are piloted in mass screening with implementation by the army in a Liverpool pilot. This is human-resource intensive, requires centralized testing centres which may be difficult to attend for all groups, and themselves may increase transmission. For reopening societies, the idea to introduce (digital) health bio-passports [5] that indicate recent COVID-19 testing status and potentially vaccination status has been floated. Such bio-passports might become the new entry passes for traveling by plane, visiting concerts, football stadiums, cinemas, etc.

## LESSONS FROM AFRICA

The continent of Africa experienced a slower start to COVID-19 and was marked by a lack of symptomatic and severe (hospitalized) cases as compared to Europe [6]. However, there are signs of increasing prevalence, eg, in Kenya where the initial urban COVID-19 epidemic is now followed by a rural one and now variants of concern are becoming a real threat. Africa has experience with many epidemics (HIV, TB, Ebola, dengue, malaria) and we argue that precious lessons can be learnt from this continent. Africa is home to various revolutionary developments, such as its leapfrogging mobile phone usage, the development of digital ‘bank-less banking’ like M-PESA [7], habituation to ubiquitous deployment of RDTs, and recent roll-out of innovative digital health care exchange platforms, such as M-TIBA [8]. Clever combination of these developments recently resulted in a successful ‘Connected Diagnostics’ approach [9]. This uses digitalized RDT results to generate semi-real time information on outbreaks, channel disease-specific funds to target populations and personalize patient interactions, thus empowering communities to better and faster access health care. Europe can truly learn from such African models.

## ROLLING OUT TESTING TO THE COMMUNITY

SARS-CoV-2 AgRDTs have recently been recommended by WHO, conditional to a sensitivity of ≥80% and a specificity of ≥97% [10]. Recent multi-centre data from FIND (Foundation for Innovative New Diagnostics) and partners indicates that achieved specificity may be even higher at >99% [11]. Application of RDTs for COVID-19 at the community level would indeed miss 10%-20% of infected individuals (false negatives), but the question is how contagious these individuals might be, since they have lower viral loads. Nowadays it is increasingly clear that 80%-90% of COVID-19 infections are caused by 10%-20% of infected patients, with super-spreaders playing an important role [12] and it is key to identify these hidden transmission events.

Today, various AgRDTs have WHO and or FDA Emergency Use qualifications. These include the STANDARD Q (SD. Biosensor, Inc) and Panbio (Abbott Rapid Diagnostics) tests which were recently jointly reserved for LMICs through the ACT-A initiative [13]. New versions of RDTs are increasingly simple, with options virus-denaturing buffers allowing for general disposal and clever digital linkages to cloud-based databases. In our view, this is more attractive than surrogate solutions for ongoing qPCR efforts, like defining viral load thresholds (or cT values cut-off values for PCR), which often differ between laboratories and test kits [14].

We argue that the global community, is ready for significant scale up of COVID-19 testing through RDTs and with increased involvement of communities. Rapid tests could be instrumental for this, since these can be performed much more regularly than PCR, therefore increasing the chance of identifying infected patients at an earlier stage, improving options for intervention. This could be realized in a stepwise manner, first targeting high-priority groups that need more frequent testing such as teachers, school children, police, in addition to symptomatic individuals in settings where laboratory testing is overloaded. Eventually, one could imagine providing RDTs through general outlets, like pharmacies. Further advances would be required like more user-friendly sample collection, ie, RDTs should be compatible with nasal and or throat sampling instead of nasopharyngeal swab. Moreover, the swab collection buffers should declare inactivation of the virus to allow for safer POC applications. Leveraging of digital tools can be particularly impactful: citizens could upload their AgRDT results through mobile phone readers (Apps) into National GDPR-databases in Africa. Incentives to do so would be priority PCR for positives, confirmation of direct contacts, and qualification for electronic vouchers to obtain new RDTs at outlets of their choice.

Thus, new COVID community data-platforms can be created at marginal costs and with little burden for health care professionals through data collected by more engaged citizens, who now will be the first to know their COVID-19 result. This will empower the community, lead to renewed engagement, and thus work as potential remedy against ‘COVID-fatigue.’ Although we acknowledge that the quality of COVID community data-platforms will be suboptimal, we argue that its sheer quantity and frequency of time-tagged and geo-tagged entries will compensate for this and provide valuable insights, just like analyses of waste water [15]. This will be needed in the time to come, given patterns of recurrent infection waves, partial vaccine implementation programs, reinfections and post-COVID syndromes.

Africa can teach the rest of the world, that there are unique possibilities to ‘democratize’ COVID-19 testing by adoption of simpler tests coupled with digital solutions, that enable decentralized approaches to engage communities. We believe that AgRDTs are sufficiently accurate for revolutionary scaled digital approaches to successfully fight COVID-19.
